# A correlation based on pressuremeter, SPT and CPT tests for characterizing of coastal alluvium: A study for phase 14 South Pars, Iran

**DOI:** 10.1016/j.mex.2022.101938

**Published:** 2022-12-05

**Authors:** Mohammad Moghadari Poor, Mohammad Azarafza, Reza Derakhshani

**Affiliations:** a Department of Civil Engineering, University of Arak, Arak, Iran; b Department of Civil Engineering, University of Tabriz, Tabriz, Iran; c Department of Earth Sciences, Utrecht University, Utrecht, Netherlands

**Keywords:** Pressuremeter test, Cone penetration test, Standard penetration test, Geotechnics, Coastal alluvium, Assalouyeh, *An empirical correlation for characterizing coastal alluvium by using PMT, SPT, and CPT tests*

## Abstract

Pressuremeter Test (PMT), Cone Penetration Test (CPT), and Standard Penetration Test (SPT) are the key in-situ experiments to directly estimate the in-situ modulus of deformation and strength parameters of soils, which are highly used in coastal alluvium. In addition, CPT and SPT are unique tests for estimating engineering properties that are ideal for onshore regions. These tests are adaptable for coastal alluvium with different saturation levels, which facilitates the determination of the field deformation modulus. Regression analysis, on the other hand, is primarily employed to estimate the empirical relationship between measured parameters and to predict geo-engineering properties. This technique is typically used to estimate the in-situ modulus of deformation and strength parameters from CPT, SPT, and PMT results. The proposed formulas in this paper used regression to correlate and characterize coastal alluvium located in phase 14 South Pars (Assalouyeh) and were compared with previously published equations. As a result of the evaluations, the correlations provided for phase 14 South Pars can be expressed as E_m_ = 0.442 q_c_ + 2.221 (R^2^ = 0.999) and P_L_ = 0.06 E_m_^0.778^ (R^2^ = 0.515).

• *This empirical method can be useful for ground assessment and estimating the in-situ modulus of deformation*.
• *This relationship can use as a modification for the original formula used based on CPT-PMT-SPT for alluvium*.
• *This empirical correlation provides fast and reliable data for Southwest Iran nearby the Persian Gulf*.

## Graphical abstract

**Figure:**
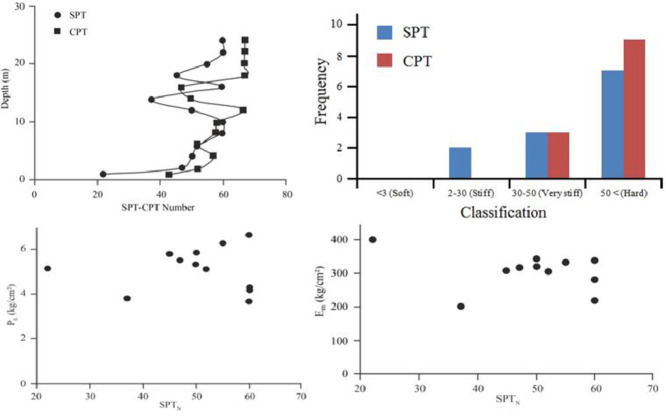

Specifications table

**Table:** 

Subject Area:	*Engineering*
More specific subject area:	*Geotechnical Engineering, Soil Mechanics*
Method name:	*An empirical correlation for characterizing coastal alluvium by using PMT, SPT, and CPT tests*
Name and reference of original method:	*Tarawneh, B., Sbitnev, A., Hakam, Y. 2018. Estimation of pressuremeter modulus and limit pressure from Cone Penetration Test for desert sands. Construction and Building Materials, 169, 299–305.*https://doi.org/10.1016/j.conbuildmat.2018.03.015*.* *Garber, J.R., Higgins, K., Meloy, N. 2018. Comparison of Direct Push to Pre-Bored Pressuremeter Test Results. ASCE IFCEE 2018, 12–22.*https://doi.org/10.1061/9780784481585.002*.* *Zhang, H., Zhang, J., Su, K., Liu, S. 2012. In-situ pressuremeter test in warm and ice-rich permafrost. Cold Regions Science and Technology 83–84, 115–121.*https://doi.org/10.1016/j.coldregions.2012.07.004*.*
Resource availability	*There are no special resources and field investigation data is presented within the article.*

## Method details

Using field or laboratory tests is the first step in identifying the geotechnical characteristics of rocks and soils [1]. Geo-engineering prefers field surveys and in-situ investigations rather than laboratory works; because field works are a direct reflection of engineering geological properties of rocks or soils that need to be characterized. By using direct information, the geo-engineers can provide an accurate and safe design [2]. To characterize engineering properties in coastal alluvium, pressuremeter (PMT), standard penetration (SPT), and cone penetration (CPT) tests have received huge attention and provide unique information about the in-situ modulus of deformation and strength parameters of soil profiles [3]. The test results are usually used for the calculation of pressuremeter modulus (E_m_), limit pressure (P_L_), cone resistance (q_c_), and sub-grade reaction modulus (K_s_) parameters. Several researchers used CPT, SPT, and PMT results to correlate the information and formulate the empirical relation between the CPT-SPT-PMT by using regression analysis to prepare more accurate results with relatively high coefficient of determination (R^2^) values [4], [5]. Table 1 provides information about the empirical relations that are estimated for E_m_ based on SPT-CPT values.

**Table 1:** A summary of experimental formulas to estimate the E_m_.

No.	Soil type	Empirical relationship	R^2^ value	Researcher(s)	Reference
SPT					
1	Sandy silty soils with clay	E_m_ = 388.67 SPT_N_ + 4554	0.83	Yagiz et al. (2008)	[6]
2	Sandy soils	E_m_ = 1.33 SPT_N_^0.77^	0.82	Bozbey and Togrol (2010)	[7]
3	Clayey soils	E_m_ = 1.61 SPT_N_^0.71^	0.72	Bozbey and Togrol (2010)	[7]
4	Clayey soils	E_m_ = 0.29 SPT_N_^1.4^	0.74	Kayabasi (2011)	[8]
5	Clayey soils	E_m_ = 1.2 SPT_N_ −3.9	0.64	Kayabasi (2011)	[8]
6	Cohesive soils	E_m_ = 1.58 SPT_N_	0.86	Balachandran et al. (2015)	[9]
7	Cohesionless soils	E_m_ = 1.09 SPT_N_	0.28	Balachandran et al. (2015)	[9]
8	Silty clay soils	E_m_ = SPT_N_ - 2. 6748	0.85	Cheshomi and Ghodrati (2015)	[10]
9	Sandy soils	E_m_ = 165.88 SPT_N_ + 1364.1	0.85	Naseem and Jamil (2016)	[11]
10	Sandy-silty clay	E_m_ = 2.611 SPT_N_ - 26.03	0.91	Özvan et al. (2018)	[12]
11	Fine-grained soils	Em = 6.4 e^0.04SPTN^	0.83	Firuzi et al. (2019)	[13]
CPT					
12	Clayey soils	E_m_ = 2.0 q_c_	0.06	Briaud et al. (1978)	[14]
13	Clayey soils	E_m_ = 2.5 q_c_	0.06	Briaud et al. (1985)	[14]
14	Sandy soils	E_m_ = 1.15 q_c_	0.06	Briaud et al. (1985)	[14]
15	Carbonate Sandy soils	E_m_ = 1.35 q_c_	–	Hamidi et al. (2000)	[15]
16	Sandy soils	E_m_ = 0.37 q_c_ + 6.5	–	Mezouar et al. (2017)	[16]
17	Desert sand	E_m_ = 0.46 q_c_ + 11.44	0.91	Tarawneh et al. (2018)	[17]

This study attempted to provide the correlated relationship by using CPT-SPT with PMT data to estimate more accurate field-based in-situ modulus of deformation and strength parameters of soils located in phase 14 of the South Pars (Assalouyeh), southwest of Iran. The South Pars is a narrow region of the foothills on the northern coast of the Persian Gulf that lies about 300 km^2^ areas and is located in Bushehr province, southwest of Iran. Geologically, the region is limited between the Persian Gulf in the south and the Assalouyeh anticline in the north. According to the stratigraphical column obtained from the South Pars region, different geological units from the late Neo-Proterozoic (Hormuz series) to Quaternary deposits (recent alluviums) are recognized in Assalouyeh. It should be noted that the previous formation of Eocene-Oligocene (Asmari) is exposed in the Assalouyeh anticline core far from the studied area [18], [19]. Phase 14 of the South Pars is located in the onshore area of the Persian Gulf on recent alluviums. Based on the ground survey and excavated boreholes, it is observed the foundations are located on an 18-meter embankment and the embankment is filled with natural sediments. The measured water-table level depth is about 3 m. Embankments are composed of a mixture of coarse-grained soils with some fine-grained along with rubble and rock fragments. At a depth of about 18 m, sandy silt and sandy gravel layers have been detected, which are related to the old alluviums (Qt^2^; Qt^2^ formations). Regarding the SPT results for natural beds, soils are classified into loose to medium-dense soils. This result for the embankment (0 m to 18 m) is classified as medium to dense soils. These results are verified based on CPT tests as well. Table 2 provides information about the CPT and SPT test results for the phase 14 site in South Pars. Of course, the application of the empirical methods to prepare the direct information about the studied site, but several limitations have to be considered during site investigations. These considerations help to provide a more accurate understanding of the site characteristics. These limitations can be classified as site uncertainties (like anisotropy in geo-units, geo-engineer experience, etc.) and device errors (due to not being calibrated, obsolete devices, and not using expert personnel). So, considering such factors can prevent calculation and execution errors.

**Table 2:** The measured CPT-SPT-PMT results for the studied site.

Depth (m)	Type of soil	SPT_N_	CPT_N_	P_L_ (kg/cm²)	E_m_ (kg/cm²)	K_s_ (kg/cm³)	q_c_
0 - 1	Silt & clay with sand & gravel	22	42	5.15	399.34	171.87	177.10
1 - 2	Gravel with silt & clayey sand	47	52	5.52	314.93	58.71	141.86
2 - 4	Gravel with silt & clay	50	57	5.85	342.65	65.00	154.36
4 - 6	Gravel with silt & clay	52	52	5.12	305.01	64.44	137.39
6 - 8	Gravel with silt & clay	60	58	4.30	218.75	50.25	98.53
8 - 10	Silt & clay with sand & gravel	60	58	4.22	281.76	52.73	126.91
10 - 12	Gravel with silt & clay	50	67	5.33	319.50	59.29	143.91
12 - 14	Gravel with silt & clay	37	50	3.82	202.88	38.25	91.38
14 - 16	Gravel with silt & clay	60	47	3.67	278.12	52.50	125.27
16 - 18	Gravel with silt & clay	45	67	5.78	308.47	58.87	138.95
18 - 20	Silt & sand with gravel	55	67	6.27	330.17	160.22	148.72
20 - 22	Silt & sand with gravel	60	67	6.63	338.10	176.04	152.29
22 - 24	Silt & sand with gravel	60	67	6.63	338.13	176.65	152.31

**Fig. 1:**
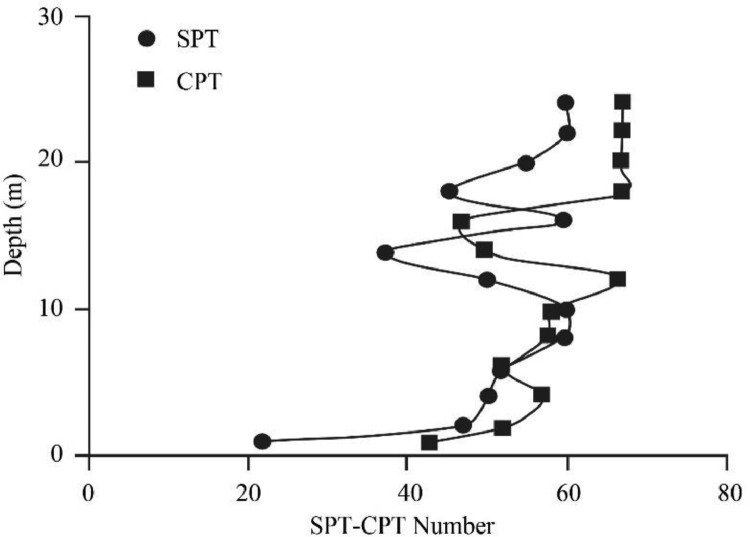
Variation of SPT_N_ CPT_N_ with depth.

**Fig. 2:**
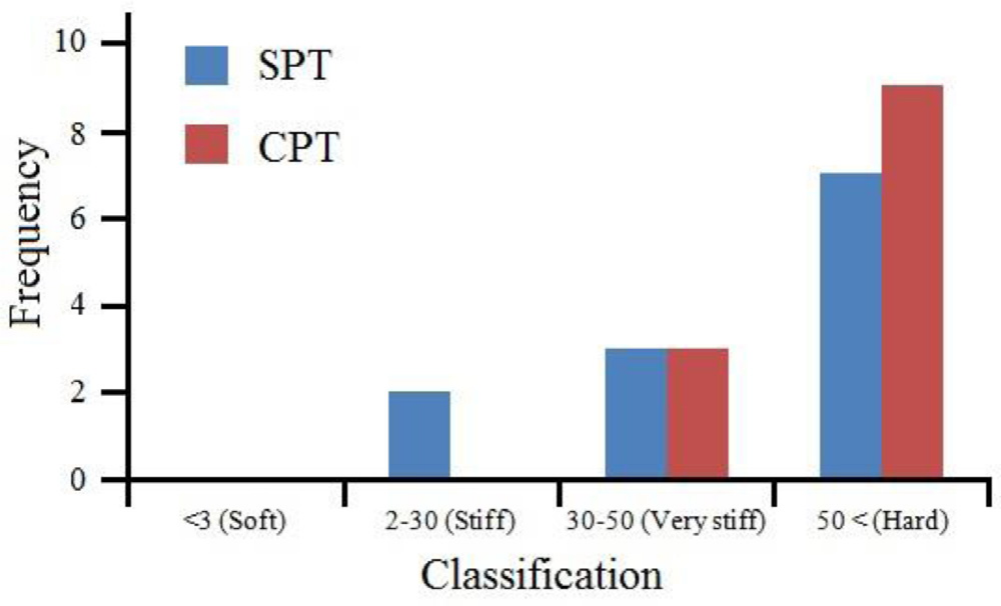
Percentage distribution and classification of the soil's strength based on CPT-SPT.

**Fig. 3:**
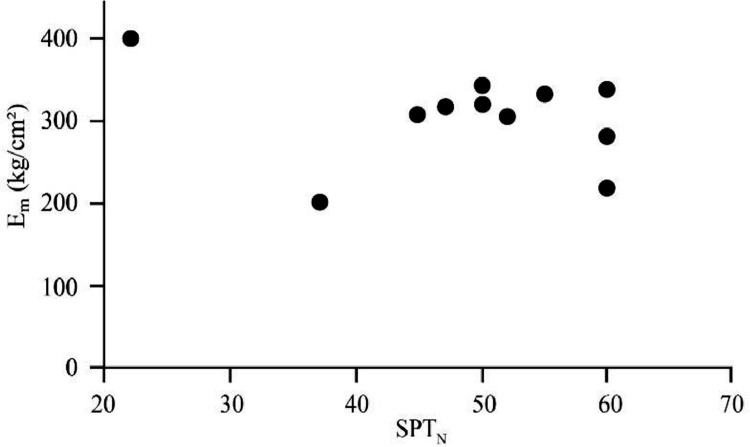
The correlation of variation for E_m_ with SPT_N_.

**Fig. 4:**
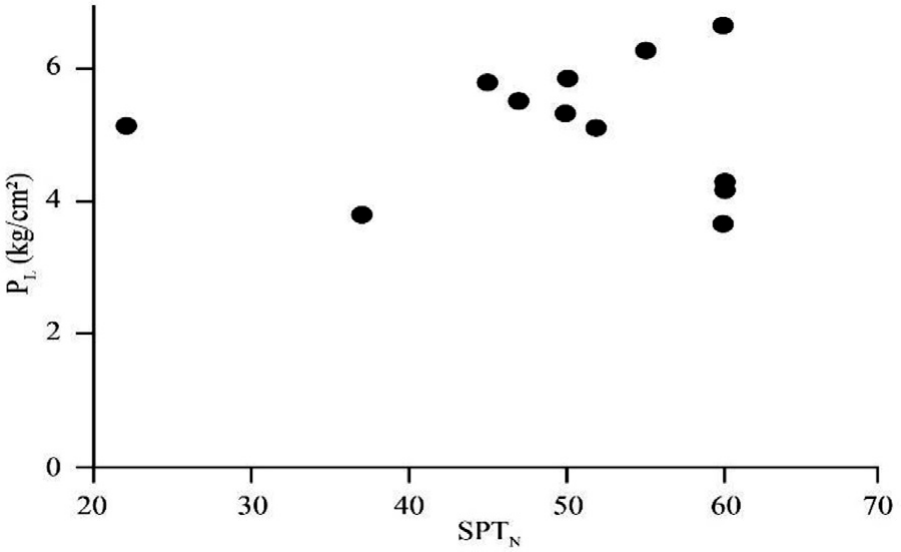
The correlation of variation for P_L_ with SPT_N_.

**Fig. 5:**
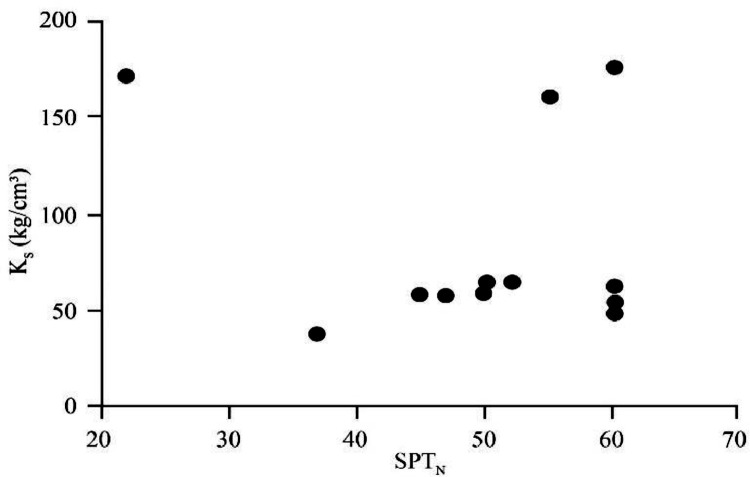
The correlation of variation for K_s_ with SPT_N_.

**Fig. 6:**
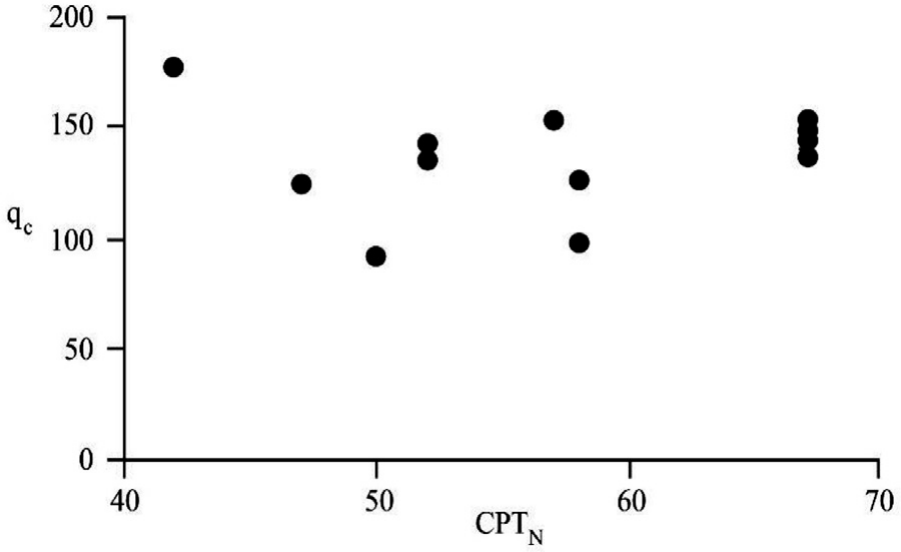
The correlation of variation for q_c_ with CPT_N_.

The performing instruction of the methods described by ASTM D1586 [20], ASTM D3441 [21], and ASTM D4719 [22]. By considering these methodologies, the variation of each index was estimated with depth. Fig. 1 presents the variation of SPT_N_ and CPT_N_ with depth in the studied site. Referring to these figures, it appears the SPT-CPT numbers vary in the 40 to 60 range. Based on these figures, the percentage distribution and classification of the soil's strength are estimated and shown in Fig. 2. Fig. 3, Fig. 4, Fig. 5, Fig. 6 provide information about the E_m_, P_L_, and K_s_ variations with SPT-CPT numbers. By conducting the regression analysis between PMT, CPT, and SPT results, the correlation of variation for parameters will be estimated, which can be expressed as Eqs. (1) to (4).

(1) $$E_{m}=0.442\;q_{c}+2.221,R^{2}=0.999$$

(2) $$P_{L}=0.060E_{m}^{0.778},R^{2}=0.515$$

(3) $$E_{m}=-1.286\;\text{SP}T_{N}+371.1,R^{2}=0.076$$

(4) $$q_{c}=0.289\text{CP}T_{N}+120.8,R^{2}=0.101$$

**Fig. 7:**
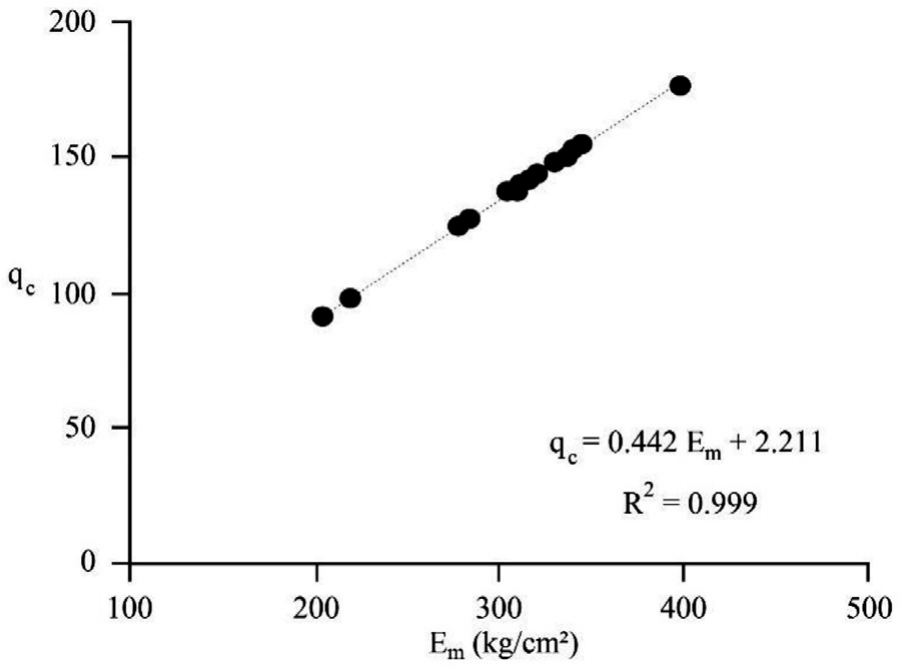
Correlation between E_m_ and q_c_.

**Fig. 8:**
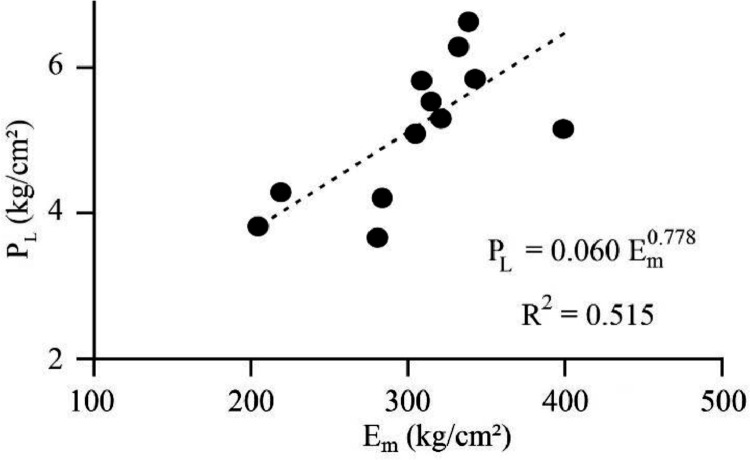
Correlation between E_m_ and P_L_.

Considering the correlation results that are presented in Eqs. (1) to (3), the variation of the E_m_ with q_c_ is provided and presented in Fig. 7. Also, Fig. 8 provides the correlation for E_m_ with P_L_ for the studied site. As seen in these figures, the R^2^ coefficient reached a considerable rate for E_m_ and a reliable rate for P_L_. Table 2

## Declaration of Competing Interest

The authors declare that they have no known competing financial interests or personal relationships that could have appeared to influence the work reported in this paper.

## Data Availability

The data that has been used is confidential.
